# Supplementary material for: Gut Ruminococcaceae levels at baseline correlate with risk of antibiotic-associated diarrhea
Source: iScience. 2021 Dec 16;25(1):103644. doi: 10.1016/j.isci.2021.103644 (PMC8718891; doi:10.1016/j.isci.2021.103644)
Supplement: Document S1. Figures S1 and S2 and Tables S1–S5 [file mmc1.pdf]

iScience, Volume *25*

Supplemental information

Gut Ruminococcaceae levels at baseline correlate with risk of antibiotic-associated diarrhea

Xiaoqiong Gu, Jean X.Y. Sim, Wei Lin Lee, Liang Cui, Yvonne F.Z. Chan, Ega Danu Chang, Yii Ean Teh, An-Ni Zhang, Federica Armas, Franciscus Chandra, Hongjie Chen, Shijie Zhao, Zhanyi Lee, Janelle R. Thompson, Eng Eong Ooi, Jenny G. Low, Eric J. Alm, and Shirin Kalimuddin

Table S1. Demographic Characteristics of Healthy Volunteers. Related to Figure 1-3. Subject 18, 28, and 32 did not meet the requirements to participate in the study during screening and were not dosed with antibiotics. Individuals with AAD are highlighted in yellow.

ID	Age	Sex	Ethnicity	Height (m)	Weight (kg)	BMI[a]	Total doses of Amoxicillin-Clavulanate
1	39	Female	Chinese	1.61	68	26.4	6
2	26	Male	Malay	1.68	71	25.0	4[c]
3	34	Female	Chinese	1.61	58	22.2	6
4	38	Male	Chinese	1.79	68	21.1	6
5	28	Male	Chinese	1.77	96	30.5	6
6	37	Male	Chinese	1.77	82	26.1	6
7	40	Female	Chinese	1.71	73	24.9	6
8	29	Female	Chinese	1.63	69	25.8	6
9	24	Female	Chinese	1.62	52	20.0	6
10	25	Male	Chinese	1.67	67	24.1	6
11	23	Female	Chinese	1.65	52	19.1	6
12	28	Female	Chinese	1.56	58	23.8	6
13	31	Female	Chinese	1.65	64	23.4	6
14	38	Female	Chinese	1.58	61	24.4	6
15	23	Female	Chinese	1.63	48	18.1	1[c]
16	29	Male	Chinese	1.6	74	28.9	5[c]
17	40	Female	Chinese	1.59	74	29.4	6
19	25	Male	Chinese	1.8	88	27.2	6
20	40	Female	Chinese	1.58	57	22.7	6
21	34	Male	Malay	1.69	68	23.9	6
22	25	Female	Chinese	1.60	74	28.9	6
23	32	Male	Chinese	1.78	108	34.1	6
24	30	Male	Chinese	1.71	73	24.8	3[c]
25	23	Male	Chinese	1.53	50	21.7	1[b]
26	24	Male	Chinese	1.75	77	25.0	6
27	23	Male	Chinese	1.72	69	23.3	6
29	24	Male	Chinese	1.77	70	22.4	6
30	40	Male	Chinese	1.76	78	25.3	6
31	30	Female	Indian	1.64	73	27.1	6
33	27	Female	Vietnamese	1.51	52	22.9	6

[b]Subject 25 developed severe vomiting after a single dose of amoxicillin-clavulanate, resulting in discontinuation of antibiotics on day 1.

[c]Subject 2, 15, 16 and 24 experienced 3 or more episodes of watery stool in a 24-hour period (Table S2), resulting in discontinuation of antibiotics.

Table S2. Demographic Characteristics of Individuals in the AAD And Non-AAD Groups. Related to Figure 1**.** Two-tailed Mann-Whitney tests were performed to test for significant differences between the AAD and non-AAD groups.

	AAD individuals (n=13)	non-AAD individuals (n=17)	p value
Sex	--	--	0.46
Female	5(38%)	10(59%)	
Male	8(62%)	7(41%)	
Age	30(26-37)	28(24-34)	0.34
BMI	24.86(23.91-26.40)	23.75(22.19-25.84)	0.39
Height	1.68(1.63-1.71)	1.63(1.59-1.75)	0.31
Weight	70.65(67.10-74.00)	68.65(57.8-73.95)	0.46

Data are n/N (%) for proportional data and median (IQR) for continuous data, unless specified otherwise.

Table S3. Frequency and Bristol Stool Scale type. Related to Figure 1-3. Faeces sample collection log book from 20 Aug 2019 to 20 Jan 2020. Subject 18, 28 and 32 failed during screening and were not dosed with antibiotics. Individuals with AAD are highlighted in yellow. Gray shaded boxes indicate that donors could not produce the stool samples or the stool samples were not collected. A total of 197 faeces samples were collected**.** BS - Bristol Stool Score; Freq – Frequency.

	Screening		Day 0		Day 1		Day 2		Day 3		Day 7		Day 14		Day 28	
ID	BS	Freq	BS	Freq	BS	Freq	BS	Freq	BS	Freq	BS	Freq	BS	Freq	BS	Freq
1	4	2	4	2	5	3	6	2	6	2	4	2	4	1	4	2
2	3	3	3	3	3	4	5	5[a]	7	8	NA	NA	3	4	4	3
3	4	1	4	1	4	1	4	1	4	2	4	2	4	1	4	2
4	3	1	3	1	3	1	3	1	4	1	3	1	4	1	NA	0
5	5	1	5	3	7	2	5	1	6	2	4	1	4	1	4	3
6	4	2	5	2	6	2	6	1	6	1	4	1	4	1	1	1
7	4	1	4	1	6	2	6	2	5	1	4	1	4	2	2	1
8	4	2	4	1	5	1	5	1	5	1	4	1	3	2	4	2
9	4	1	4	1	4	1	5	1	5	2	4	1	3	1	4	2
10	4	2	4	1	4	1	4	1	7	4	NA	NA	NA	NA	4	4
11	3	1	4	1	4	1	4	2	4	1	2	1	3	1	4	1
12	4	1	4	1	NA	NA	NA	NA	4	1	4	1	4	1	4	1
13	5	1	6	1	6	1	5	1	4	2	6	2	4	1	6	2
14	3	1	4	1	4	1	4	2	4	1	4	2	4	2	3	1
15	3	1	3	1	7	3[b]	6	3	4	1	5	1	4	2	3	1
16	4	1	6	1	6	1	6	2	7	5[c]	6	1	6	2	6	1
17	4	1	4	2	4	1	4	1	4	1	4	1	4	1	4	1
19	6	1	3	2	7	2	2	1	3	1	2	1	4	1	3	1
20	3	1	4	1	6	1	6	1	3	1	3	1	3	1	4	2
21	2	1	4	1	4	2	6	1	3	1	4	1	3	2	6	1
22	5	1	6	1	4	1	4	1	4	1	4	1	4	1	4	2
23	4	1	3	2	4	1	4	1	4	1	4	2	4	1	4	2
24	5	2	5	1	7	3[d]	4	2	1	2	4	2	4	1	4	2
25	3	1	3	1	4	1	5	1	4	1	5	1	4	1	4	1
26	4	1	3	2	3	1	4	2	4	2	4	2	4	2	4	2
27	4	1	4	2	5	1	5	3	5	1	4	1	4	2	4	1
29	4	1	4	2	4	2	4	3	4	2	5	2	4	2	4	3
30	4	1	4	2	2	1	2	2	2	1	5	4	4	1	4	1
31	4	3	4	4	4	2	4	2	4	2	4	2	5	1	4	2
33	2	1	1	2	2	1	3	1	2	1	1	1	NA	NA	NA	NA

[a]Subject 2 experienced 5 episodes of watery stool in a 24-hour period, resulting in discontinuation of antibiotics on day 2, with the total doses of amoxicillin-clavulanate as 4.
[b]Subject 15 experienced 3 episodes of watery stool in a 24-hour period, resulting in discontinuation of antibiotics on day 1, with the total doses of amoxicillin-clavulanate as 1.
[c]Subject 16 experienced 5 episodes of watery stool in a 24-hour period, resulting in discontinuation of antibiotics on day 3, with the total doses of amoxicillin-clavulanate as 5.
[d]Subject 24 experienced 3 episodes of watery stool in a 24-hour period, resulting in discontinuation of antibiotics on day 1, with the total doses of amoxicillin-clavulanate as 3.

Table S4. Primer-probe sets used in this study. Related to Figures 2-4**.**

Name	Sequences	References
16S_V4_515F primer	GTGCCAGCMGCCGCGGTAA	Caporaso et al., 2011
16S_V4_806R primer	GGACTACHVGGGTWTCTAAT	
F. prau_FP	GATGGCCTCGCGTCCGATTAG	Carroll et al, 2012
F. prau_RP	CCGAAGACCTTCTTCCTCC	
16S_FP	ACTCCTACGGGAGGCAGCAG	Van Hul et al, 2020
16S_RP	ATTACCGCGGCTGCTGG	
TcdA_FP	CAGTCGGATTGCAAGTAATTGACAAT	Kubota et al, 2014
TcdA_RP	AGTAGTATCTACTACCATTAACAGTCTGC	
TcdA_Probe	/56-FAM/TTGAGATGATAGCAGTGTCAGGATTG/36-TAMSp/	
TcdB_FP	TACAAACAGGTGTATTTAGTACAGAAGATGGA	
TcdB_RP	CACCTATTTGATTTAGMCCTTTAAAAGC	
TcdB_Probe	/56-FAM/TTTKCCAGTAAAATCAATTGCTTC/36-TAMSp/	

Table S5. gBlocks Gene Fragments sequences for *Faecalibacterium prausnitzii,*16S rRNA, *C. difficile* TcdA and TcdB gene. Related to Figure 4.

gBlocks Gene Fragments	Sequences
gBlocks Gene Fragments for *Faecalibacterium prausnitzii* and 16S rRNA positive control	GGTAGAGGGAAAAGGAGCAATCCGCTTTGAGATGGCCTCGCGTCCGATTAGCTAGTTGGTGAGGTAATGGCCCACCAAGGCGACGATCGGTAGCCGGACTGAGAGGTTGAACGGCCACATTGGGACTGAGACACGGCCCAGACTCCTACGGGAGGCAGCAGTGGGGAATATTGCACAATGGGGGAAACCCTGATGCAGCGACGCCGCGTGGAGGAAGAAGGTCTTCGGATTGTAAACTCCTGTTGTTGAGGAAGATAATGACGGTACTCAACAAGGAAGTGACGGCTAACTACGTGCCAGCAGCCGCGGTAATACGTAGGTCACAAGCGTTGTCCGGAATTAC
gBlocks Gene Fragments for *C. difficile* TcdA positive control	GGTAGAGGGAAAAGGAGCAATCCGCTTTGAGATGGCCTCGCGTCCGATTAGCTAGTTGGTGAGGTAATGGCCCACCAAGGCGACGATCGGTAGCCGGACTGAGAGGTTGAACGGCCACATTGGGACTGAGACACGGCCCAGACTCCTACGGGAGGCAGCAGTGGGGAATATTGCACAATGGGGGAAACCCTGATGCAGCGACGCCGCGTGGAGGAAGAAGGTCTTCGGATTGTAAACTCCTGTTGTTGAGGAAGATAATGACGGTACTCAACAAGGAAGTGACGGCTAACTACGTGCCAGCAGCCGCGGTAATACGTAGGTCACAAGCGTTGTCCGGAATTAC
gBlocks Gene Fragments for *C. difficile* TcdB positive control	TACACCCCTGCGGCAACGTTGAAGCTCCTGGATTACACTGGCTGGATCTAAGCCGTGACACCCGTCATACTCCATAACCGTCTGTAACTCACGGCTTGTTCTGGACTGGATTGCCATTCTCTCAGAGTATTATGCAGGCCGGCGTACGGGTCCCATATAAACCTGTCATAGCTTACCTGACTCTACTTGGAAATGTGGCTTACAAACAGGTGTATTTAGTACAGAAGATGGATTTAAATATTTTGCCCCAGCTAATACACTTGATGAAAACCTAGAAGGAGAAGCAATTGATTTTACTGGAAAATTAATTATTGACGAAAATATTTATTATTTTGATGATAATTATAGAGGAGCTGTAGAATGGAAAGAATTAGATGGTGAAATGCACTATTTTAGCCCAGAAACAGGTAAAGCTTTTAAAGGTCTAAATCAAATAGGTG

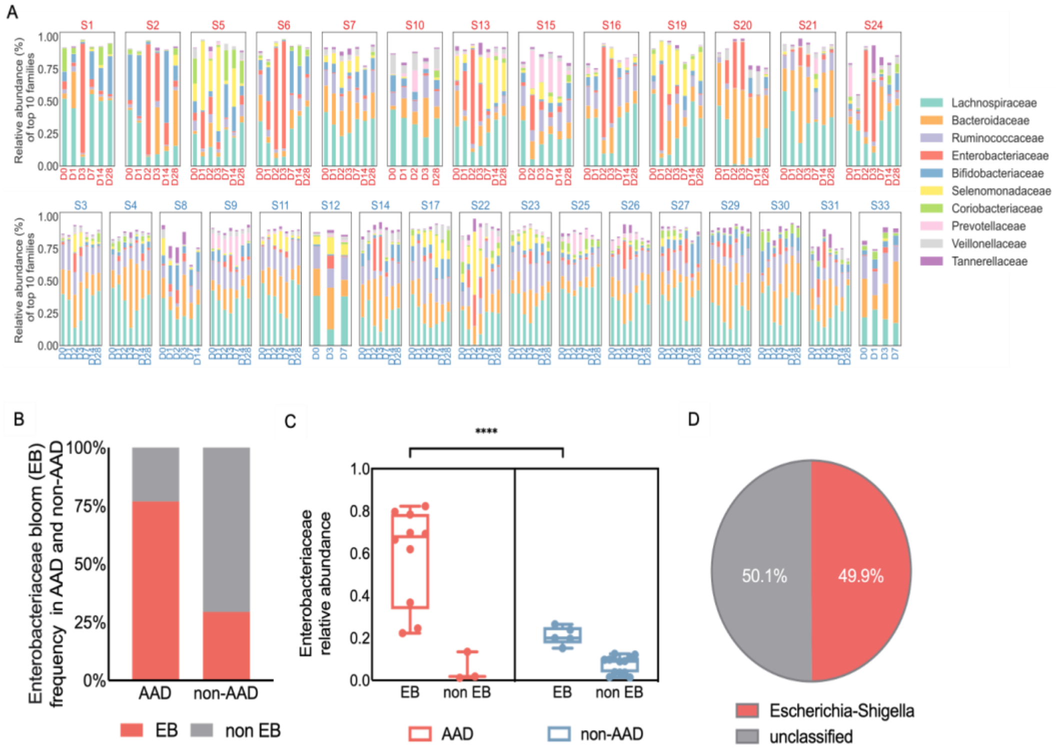

Figure S1. Enterobacteriaceae bloom (EB) were more common in the AAD group with a much higher magnitude.

Related to Figure 2. (A) A panel list of the 10 most abundant families in individuals in the AAD (top panel) and non-AAD groups (bottom panel). (B) Enterobacteriaceae blooms (EB) were observed more frequently in the AAD group compared to the non-AAD group (76.9% vs 29.4%). As the increase in Enterobacteriaceae varies between days 1-3 across different individuals, we defined Enterobacteriaceae blooms as having the maximum of Enterobacteriaceae abundance across days 1-3 in each individual being larger than the median(max(days 1-3)-baseline). (C) For all the individuals who experienced EB, the relative abundance of Enterobacteriaceae is higher in the individuals with AAD compared to the individuals with non-AAD (mean 0.59 vs 0.21, FDR corrected $p < 0.0001$). (D) Pie charts of the most abundant genera in Enterobacteriaceae across days 1-3. Within the Enterobacteriaceae family, we observed a higher abundance of Escherichia-Shigella (49.9%) at the genus level.

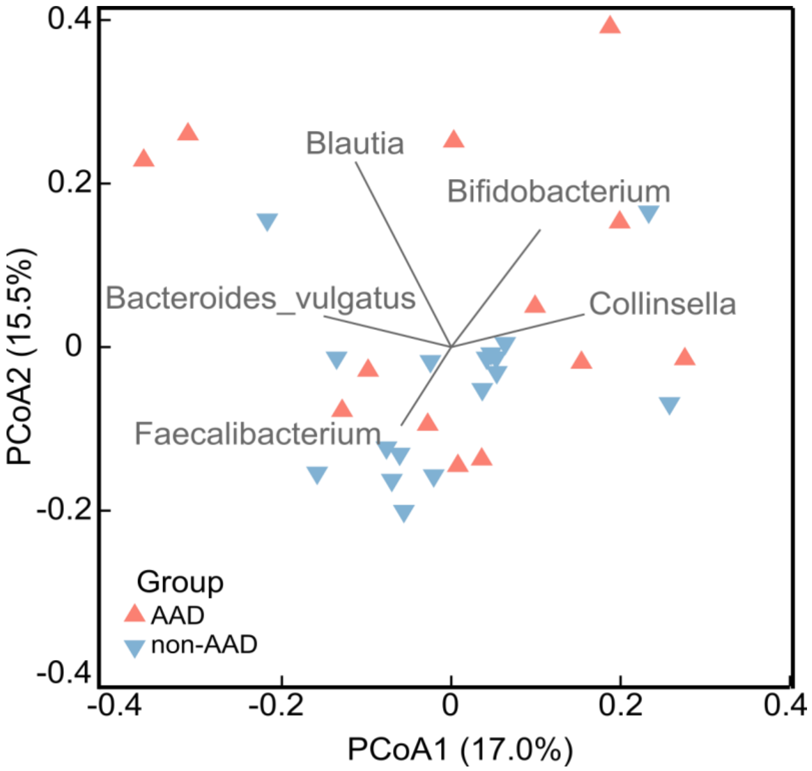

Figure S2. PCoA of baseline samples based on the ASV level bray-curtis dissimilarity. Related to Figure 3. Display is based on sample scores on the primary axis (PCoA1, 17.0% variance explained) and secondary axis (PCoA2, 15.5% variance explained). Vector loading plots at the ASV level indicate that ASV assigned to *Faecalibacterium* points to the non-AAD group while many ununified features (e.g., ASVs assigned to Blautia, Bifidobacterium, Collinsella, Bacteroides vulgatus) point to the AAD groups (multiple linear correlation with the threshold setting as 0.3).
